# A tissue-based approach to selection of reference genes for quantitative real-time PCR in a sheep osteoporosis model

**DOI:** 10.1186/s12864-017-4356-4

**Published:** 2017-12-19

**Authors:** Felix Schulze, Deeksha Malhan, Thaqif El Khassawna, Christian Heiss, Anja Seckinger, Dirk Hose, Angela Rösen-Wolff

**Affiliations:** 1 Department of Pediatrics, University Hospital Carl Gustav Carus, TU Dresden, Fetscherstraße 74, 01307 Dresden, Germany; 2 Experimental Trauma Surgery, Justus-Liebig University, Aulweg 128, 35392 Giessen, Germany; 3 Department of Trauma, Hand and Reconstructive Surgery, University Hospital of Giessen-Marburg, Rudolf-Buchheim-Strasse 7, 35385 Giessen, Germany; 4 Labor für Myelomforschung, Medizinische Klinik V, Universitätsklinikum Heidelberg, Im Neuenheimer Feld 410, 69120 Heidelberg, Germany

**Keywords:** Reference gene, Sheep, MSC, geNorm, NormFinder, BestKeeper, Delta Ct method

## Abstract

**Background:**

In order to better understand the multifactorial nature of osteoporosis, animal models are utilized and compared to healthy controls. Female sheep are well established as a model for osteoporosis induced by ovariectomy, calcium and vitamin D low diet, application of steroids, or a combination of these treatments. Transcriptional studies can be performed by applying quantitative real time PCR (RT-qPCR). RT-qPCR estimates mRNA-levels of target genes in relation to reference genes. A chosen set of reference genes should not show variation under experimental conditions. Currently, no standard reference genes are accepted for all tissue types and experimental conditions. Studies examining reference genes for sheep are rare and only one study described stable reference in mandibular bone. However, this type of bone differs from trabecular bone where most osteoporotic fractures occur. The present study aimed at identifying a set of reference genes for relative quantification of transcriptional activity of ovine spine bone and ovine in vitro differentiated mesenchymal stromal cells (MSC) for reliable comparability.

**Methods:**

Twelve candidate reference genes belonging to different functional classes were selected and their expression was measured from cultured ovMSCs (*n* = 18) and ovine bone samples (*n* = 16), respectively. RefFinder was used to rank the candidate genes.

**Results:**

We identified *B2M*, *GAPDH*, *RPL19* and *YWHAZ* as the best combination of reference genes for normalization of RT-qPCR results for transcriptional analyses of these ovine samples.

**Conclusion:**

This study demonstrates the importance of applying a set of reference genes for RT-qPCR analysis in sheep. Based on our data we recommend using four identified reference genes for relative quantification of gene expression studies in ovine bone or for in vitro experiments with osteogenically differentiated ovine MSCs.

**Electronic supplementary material:**

The online version of this article (10.1186/s12864-017-4356-4) contains supplementary material, which is available to authorized users.

## Background

In order to better understand the multifactorial nature of osteoporosis, small and large animal models are utilized upon the recommendations of the Food and Drug Administration (FDA). Female sheep are well established as model for osteoporosis as their macro- as well as microarchitecture is comparable to human bone [1]. Osteoporosis in sheep is induced by ovariectomy, calcium and vitamin D low diet, application of steroids, or a combination thereof [2, 3].

Besides recent advances, e.g. in terms of using RNA-sequencing or DNA-microarrays, quantitative real time PCR (RT-qPCR) is still used for quantifying mRNA to reflect relative expression of genes of interest (GOI) or to evaluate results obtained from RNA-sequencing. This is a powerful tool because of its high sensitivity, specificity, and low cost. Quantification of gene expression is impacted amongst other factors by the variability of starting material in terms of cell type, RNA recovery and integrity and efficiency of cDNA synthesis [4]. One way to compensate for this is normalization to constitutively expressed genes without major variation across different experimental conditions in the respective tissues, i.e. so-called reference genes [5, 6].

For quantification of gene transcription, previous studies had shown the most suitable reference genes for ovine spleen, lung, ileum, pulmonary and nervous tissue and blood [7–11]. Only one bone-related study investigated reference genes in ovine mandibular condyle [12]. However, the type of mandibular bone is different from trabecular bone in the spine and long bones where most osteoporotic fractures occur [13]. For a better reproducibility of ovine bone samples from an osteoporosis model, we used differentiated ovine bone marrow MSCs for comparison. Ovine bone samples derived from osteoporosis models must be compared to control ovine bone samples. In addition these samples should be compared to in vitro osteogenically differentiated ovine bone marrow MSCs in order to evaluate the results.

Aim of the present study was to identify a reliable set of RT-qPCR reference genes for quantification of relative expression changes in pathological ovine bone samples. By using twelve PCR assays for assessment of potential reference genes belonging to different functional classes, cell specific stable expression was first determined in cultures of ovine MSCs (ovMSCs) after in vitro differentiation into osteoblasts. Second, tissue specific stable expression was determined by investigating ovine bone from both control and osteoporotic sheep. Suitability of reference genes were assessed using RefFinder [14]. This tool combined the delta Ct, Normfinder, Bestkeeper and GeNorm algorithm, to calculate a final overall ranking. For a better understanding of identified reference genes, gene ontology and netword analysis using NCBI-DAVID [15] as well as GeneMANIA [16] and Cytoscape [17], respectively, were performed.

## Methods

### In vitro / cell culture

ovMSCs from bone marrow of healthy sheep were cultured in α-MEM supplemented with 10% FCS, L-glutamine and pen/strep mixture (Biochrom GmbH, Berlin, Germany) with medium changes every 3–4 days. Cells were seeded in T150 flask and incubated at 37 °C, 5% CO_2_ in a humidified atmosphere until reaching 80–90% confluence. For osteoblast differentiation ovMSCs (at passage 3) were seeded at a density of 2 × 10^6^ cells per T150 flask, medium was supplemented with 10 × 10^−7^ M dexamethasone, 3 mM NaH_2_PO_4_ and 0.05 mM ascorbic acid 2-phoshate (Sigma Aldrich, Taufkirchen, Germany) and the medium was changed twice a week. Osteogenic differentiation was controlled by AlizarinRed S (40 mM) (Sigma Aldrich, Taufkirchen, Germany) staining at day 7, d14, d21 and d28 (Additional file 1).

### RNA isolation from ovMSCs

After day 0, d7, d14, d21, and d28 (control and osteogenic differentiation, respectively), cells were collected by scraping in PBS and centrifuged at 400 g for 5 min. RNA was extracted using the SV total RNA extraction kit (Promega GmbH, Mannheim, Germany) according to the manufacturer’s instructions (including DNAseI digestion).

### Osteoporosis induction in sheep model

Thirty one female Merino sheep with an average age of 5.5 years were utilized for this study. Animals were purchased from different farmers after being sorted for meat production. The sheep received a clean bill of health by a veterinarian. The owners agreed to the use of animals in research purposes and were shortly advised about the nature of the research and its impact on clinical translation.

This study was performed in accordance with the Institutional and German animal protection laws and approved by the ethical commission of the local governmental institution (“Regierungspräsidium Darmstadt”, permit no. Gen. Nr. F31/36). Animals were randomly divided into 4 groups: control group (Control, 0 M, *n* = 8), bilaterally ovariectomized group (OVX, *n* = 7), bilaterally ovariectomized and treated with diet deficient of calcium and vitamin D3 (OVXD, *n* = 8) and triple treatment group; in addition to the treatment received by ovx + diet + steroids animals, this group also received a biweekly dosage of glucocorticoid (methylprednisolone) (OVXDS, *n* = 8) (Table 1). Bone samples of four animals per group were used in the present study and combined for analyses. Animals received a premedication of 10 mg/kg ketaminhydrochloride (Ketavet® 10 mg/mL, Bela- Pharm GmbH und Co.KG, Germany), 0.01 mL/kg xylazin (Rompun® 2%, Bayer AG, Germany), 0.3 mg/kg midazolam (Midazolam Rotexmedica 5 mg/mL, Rotexmedica GmbH, Germany), 0.01 mg/kg atropine (Atropinsulfat 0.5 mg/mL, B. Braun Melsungen AG, Germany)) for anesthesia prior to bilateral ovariectomy or sham operations, respectively. Subsequently intravenous anesthesia was administered with 2 mg/kg propofol (Propofol 2% (20 mg/1 mL), Fresenius Kabi, Germany) and 2 μg/kg fentanyl (Fentanyl-Hameln 50 μg/mL, Hameln pharmaceuticals GmbH, Germany).

**Table 1:** Overview of interventions in sheep osteoporosis model

	Surgery	Medication	Antibiotic	Diet	Cortico-steroids
Control	Sham	X	X	standard feed	–
OVX	ovariectomy	X	X	standard feed	–
OVXD	ovariectomy	X	X	deficient in calcium and vitamin D3	–
OVXDS	ovariectomy	X	X	deficient in calcium and vitamin D3	X

Animals were treated with the respective procedures for eight months

*OVX* ovariectomy, *OVXD* ovariectomy + diet, *OVXDS* ovariectomy + diet + steroids

After surgery, animals received 0.01 mg/kg buprenorphinhydrochlorid (TEMGESIC® ampoules 0.3 mg, RB Pharmaceuticals GmbH, Germany) subcutaneously twice daily for analgesia of as well as 0.5 mg/kg meloxicam (Metacam® 20 mg/mL ad. us. vet., Boehringer Ingelheim Vetmedica GmbH, Germany) as non-steroidal anti-inflammatory drug once a day intramuscularly. Opiates were reduced gradually at the discretion of the veterinarians. In the first five days post-surgery, animals were injected intramuscularly with antibiotics, 0.1 mL/kg penicillin (Veracin® RS, Albrecht GmbH, Germany), every 48 h.

#### Animal diet

The experimental groups OVXD and OVXDS received a diet deficient in calcium and vitamin D3 compared to standard diet (Cat No S6189-S010, Sondermischung Schaf, 4 mm pellet, SNIFF Spezialdiäten GmbH, Germany) twice daily. In addition, animals had access to straw ad libitum. The other two groups, i.e. Control and OVX, were fed with standard feed (SNIFF Spezialdiäten GmbH, Germany) during wintertime while being held on a pasture in spring.

#### Administration of corticosteroids

Two weeks after ovariectomy, sheep in the OVXDS group received 320 mg methylprednisolone/sheep (Depot- Medrate® ad us. vet 40 mg/mL intramuscular injection suspension, Pfizer Deutschland GmbH, Germany) every 14 days.

#### Euthanasia

After eight months, animals were euthanized by intravenous administration of 50 mg/kg pentobarbital (Anestesal®, Pfizer, Mexico) under anesthesia as described above.

Bone biopsies were collected from the 5th lumar spine using a trephine (5 mm diameter, Meisinger, Neuss, Germany), directly frozen in liquid nitrogen and kept in −80 °C until RNA extraction.

### RNA isolation from bone

Ovine bone samples were crushed and milled in liquid nitrogen using pestle and mortar (Morgan Advanced Materials Haldenwanger GmbH, Waldkraiburg, Germany), until a fine powder was observed. Afterwards the bone powder was transferred to a 2 mL tube and 1 mL Trizol (LifeTechnologies, Darmstadt, Germany) was added. The mixture was homogenized with an UltraTurrax T10 (IKA GmbH, Königswinter, Germany) for 30s.

This suspension was then centrifuged at 400 g for 5 min at 4 °C to separate lipid debris. The clear Trizol mixture was transferred into a new tube and 200 μL chloroform was added and incubated for 15 min at room temperature. Phase separation was performed by centrifugation at 11,500 g for 15 min at 4 °C. The aqueous phase was transferred into a new 2 mL tube and the same volume of 70% EtOH was added before transferring to the RNaesy spin column (Qiagen, Hilden, Germany). The RNaesy protocol was followed as per the manufacturer’s instructions including DNAseI digestion (RNAse free DNAse set, Qiagen, Hilden, Germany).

RNA concentration was measured using Quantifluor RNA system (Promega GmbH, Mannheim, Germany) and the Quantus Fluorometer.

### RNA quality analysis

RNA quality control was performed using an Agilent 2100 Bioanalyzer (Agilent, Böblingen, Germany) according to the manufacturers’ instructions. RNA quality is controlled by comparing the 28S to 18S RNA peak ratio (2:1 in non-degraded RNA-samples) or the so-called RNA-integration number (RIN). RIN values above 6.5 were accepted and RNA was used for further analysis.

### Gene selection and primer design

Twelve genes were selected which are commonly used as reference genes from various functional classes, i.e. *GAPDH, ALAS1, HPRT, EF-2, G6PDH, ACTB, RPL19, B2M, YWHAZ, SDHA, PGK1,* and *TFRC* (Eurofins Genomics, Ebersberg, Germany). Primers were designed using Primer3 software via PrimerBlast (NCBI) and selected to produce amplicons spanning two exons; specificity was validated using cDNA from normal cultured ovMSCs in endpoint PCR assays (Table 2). PCR products were separated on a 2.5% agarose gel to validate expected size.

**Table 2:** Primer list and sequences of 12 candidate reference genes for real-time PCR

Gene name	Gene product	Function	Accession number	Primer sequence	Primer efficiency
*GAPDH* (98 bp)	Glyceraldehyde 3-phosphate dehydrogenase	Enzyme of glycolysis	NM_001190390.1	F:5’ ACAGTCAAGGCAGAGAACGG 3′ R:5’ CCAGCATCACCCCACTTGAT 3’	97.0%
*ALAS1* (123 bp)	Delta-aminolevulinate synthase 1	Enzyme of heme biosynthetic pathway	XM_004018407.3	F:5’ CACTGCCCCAGTCACATCAT 3′ R:5’ GGGCACTGTGGGGTAATTGA 3’	95.5%
*HPRT* (102 bp)	Hypoxanthine-guanine phosphoribosyl-transferase	Enzyme of purine salvage pathway	XM_015105023.1	F:5’ TTCTTTGCCGACCTGTTGGA 3′ R:5’ TCACCTGTTGACTGGTCGTT 3’	92.5%
*EF-2* (106 bp)	Elongation factor 2	required for the translocation step in protein synthesis	XM_012178077.2	F:5’ GTTGTGAAGGCCTACCTCCC 3′ R:5’ GCCAGTGGTCAAACACACAC 3’	99.5%
*G6PDH* (106 bp)	Glucose-6-phosphate dehydrogenase	Enzyme of pentose phosphate pathway	NM_001093780.1	F:5’ ATTGTGGAGAAGCCCTTCGG 3′ R:5’ GGTAGTGGTCGATGCGGTAG 3’	94.5%
*ACTB* (95 bp)	Beta-actin	Cytoskeletal structural protein	NM_001009784.2	F:5’ GCAGATGTGGATCAGCAAGC 3′ R:5’ GGGTGTAACGCAGCTAACAG 3’	92.0%
*RPL19* (126 bp)	Ribosomal protein L19	Ribosomal protein	XM_004012836.2	F:5’ AGCCTGTGACTGTCCATTCC 3′ R:5’ ACGTTACCTTCTCGGGCATT 3’	99.0%
*B2M* (91 bp)	Beta-2 microglobulin	Beta-chain of MHC-I	NM_001009284.2	F:5’ CCTTGGTCCTTCTCGGGCTG 3′ R:5’ TCTGGCGGGTGTCTTGAGTAT 3’	98.5%
*YWHAZ* (124 bp)	Tyrosine 3-monooxygenase/ tryptophan 5-monooxygenase activation protein, zeta	Signal transduction	NM_001267887.1	F:5’ GATGAAGCCATTGCTGAACTTGA 3′ R:5’ CAGCTTCGTCTCCTTGGGTA 3’	97.0%
*SDHA* (104 bp)	Succinate dehydrogenase	Enzyme of mitochondrial respiratory chain	XM_012097183.1	F:5’ GAGTTCGTGCAGTTCCACCC 3′ R:5’ CTCTCACCCTGGCTGTTGAT 3’	91.5%
*PGK1* (104 bp)	Phosphoglycerate kinase 1	Enzyme of glycolysis	NM_001142516.1	F:5’ CCTCTGGCATACCTGTTGGC 3′ R:5’ CACCCACAGGTCCATTCCAC 3’	92.5%
*TFRC* (96 bp)	Transferrin receptor	Transmembrane glycoprotein	XM_004003001.2	F:5’ ACCTCAAATCAGCGCTGTCA 3′ R:5’ CAGCCTCACGTGGGACATAA 3’	93.5%

### cDNA synthesis and RT-qPCR

cDNA synthesis was performed using M-MLV reverse transcriptase (Promega GmbH, Mannheim, Germany). Briefly, 1 μg RNA was mixed with 0.5 μL random hexamer (10 μM) and 0.5 μL oligo dT primer (10 μM) and filled up with H_2_O to 25.4 μL reaction volume. After incubation at 70 °C for 10 min and chilling at 4 °C for 5 min, the reverse transcriptase mixture including buffer, dNTPs, RNAsin Plus (Promega GmbH, Mannheim, Germany) and MMLV-RT –H was added (complete reaction volume 35 μL). Transcription was performed at 42 °C for 60 min and inactivated at 95 °C for 5 min. Obtained cDNA was stored at −20 °C until use.

For RT-qPCR GoTaq qPCR Master Mix (Promega GmbH, Mannheim, Germany) was used according to the manufacturer’s instructions with primer concentrations of 0.25 μM. RT-qPCR was run at the Applied Biosystems 7300 Real-Time PCR System using the following cycling parameters: 95 °C 10 min, 40 cycles of 60 °C 1 min and denaturation at 95 °C for 30s for all primer pairs. Afterwards melting curve was analyzed to determine specificity of reaction products.

### Data analysis

RT-qPCR data of candidate genes were analyzed for stable expression using the online tool RefFinder (http://leonxie.esy.es/RefFinder/). The tool encompasses GeNorm, Normfinder, Bestkeeper and delta Ct algorithm which allows for comparing and ranking of experimental candidates. The delta Ct method [18] compares differences of Ct values by comparing two reference genes pairwise. The candidate reference genes are ranked according to the mean standard variation of the mean delta Ct differences. If the delta Ct values of pairs are constant for all tested samples, these genes are considered to have stable expression or they are regulated in the same way. The Bestkeeper software [19] performs a pairwise correlation of the Ct values in each pair of candidates. It is based on the fact that the best gene has the lowest Ct variation when the cDNA input is constant. The analysis is based on the standard deviation and coefficient of correlation of all tested genes. Standard deviation values were obtained from the Ct values of each tested gene and the correlation coefficients were calculated using Pearsons pair-wise correlation analyses between each gene and the geometric mean of the Ct values. Genes with high standard variation considered being unreliable and the remaining genes are ranked according to their correlation coefficient. Normfinder [20] calculates variations between sample groups. Therefore, inter- and intra-group expression variation was analyzed and the stability value (σ-value) was then calculated and ranked based on gene expression profiles. The last algorithm GeNorm [4] calculates the average pairwise variation of each reference gene from all other reference gene candidates (M-value). It performs a ranking of the candidate genes by stepwise exclusion of the worst scoring gene. Then calculation of the average gene expression stability is repeated. At the end two genes are detected which have the most stable gene expression.

Based on the rankings of each algorithm, the tool calculates geometric means in order to create a final overall ranking. The analysis contains ovMSCs, 2 probes of each day 0, 7, 14, 21, and 28 of both control and osteogenic differentiation and from the animal model 4 samples of each treatment (control, OVX, OVXD and OVXDS). Both models in vitro (*n* = 18) and in vivo (*n* = 16) were analyzed separately as well as combined.

To better understand the reference genes, gene ontology classification using NCBI-DAVID [15] was performed. To further understand the interaction between the reference genes, network analysis using GeneMANIA [16] and Cytoscape [17] were performed.

## Results

Primer pairs were designed to amplify a specific gene product which resulted in a single peak in melt curve analysis, and the expected amplicon size on agarose gel. Ubiquitous expression of tested reference genes was considered by BioGPS sheep gene expression atlas dataset [21]. Results are shown in Additional file 2 and revealed that all of these genes were expressed. Ct values and thus expression of genes differed between in vitro differentiated ovMSCs and bone tissue.

In bone samples *ACTB* (mean Ct ± SD 17.21 ± 1.77), *RPL19* (17.28 ± 0.91) and *B2M* (17.59 ± 1.06) were expressed prevalently, whereas *TFRC* (22.10 ± 2.75), *G6PDH* (22.71 ± 2.03), and *SDHA* (23.92 ± 1.69) were expressed rarely across all four groups (control, OVX, OVXD and OVXDS).

**Fig. 1:**
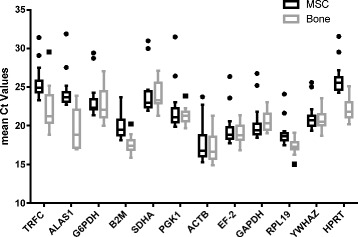
Boxplots of real time PCR Ct values of all candidate genes tested. Values are given as the real-time PCR threshold value (Ct) from ovMSCs, two sets of control and osteogenically differentiated ovMSCs were analyzed on days 0, 7, 14, 21 and 28 and the resulting data were combined. Four samples of ovine bone originating from animals that had been treated by sham (control), ovariectomy (OVX), ovariectomy + diet (OVXD) or ovariectomy + diet + steroids (OVXD), respectively, were analyzed and the resulting data were combined

In osteogenically differentiated ovMSCs (day 0, 7, 14, 21 and 28) the highest expression was found for *ACTB* (17.77 ± 2.39), *RPL19* (18.96 ± 1.56), and *EF-2* (19.61 ± 2.15), the lowest expression for *ALAS1* (24.31 ± 2.20), *TFRC* (25.51 ± 2.03), and *HPRT* (25.99 ± 1.88) (Fig. 1). To evaluate the most stable expressing reference genes, we used the online available tool RefFinder.

### Application of the delta ct method

**Fig. 2:**
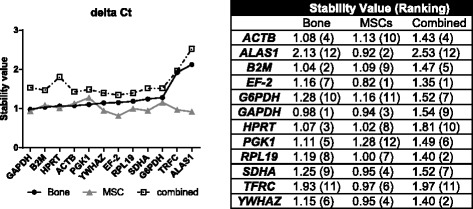
Ranking of candidate reference genes by delta Ct method in cells and tissue. Candidate reference genes were ranked by their stability value, as calculated by the delta Ct method. MSCs were both, osteogenic differentiated and control cells from ovMSCs at different time points (day 0, 7, 14, 21, and 28). Bone samples related to a pool of all experimental groups (control, OVX, OVXD and OVXDS). These samples were used for the consecutive arrangement of the graph

The results of the delta Ct method are shown in Fig. 2. Based on this analysis, *GAPDH* (stability value = 0.98) and *B2M* (1.04) were the most stable reference genes in ovine bone. The groups of bone tissue in this study consisted of samples from control and different osteoporosis induction treatment (control, OVX, OVXD & OVXDS).

In differentiated ovMSCs *EF-2* (0.82) was the most stable gene followed by *ALAS1* (0.92). The combined group identified *EF-2* (1.35), *RPL19* (1.40), and *YWHAZ* (1.40) as best reference genes. *ALAS1* was the least stable gene in the combined group, although it showed the second lowest stability value of the ovMSCs group.

### Application of the Bestkeeper algorithm

**Fig. 3:**
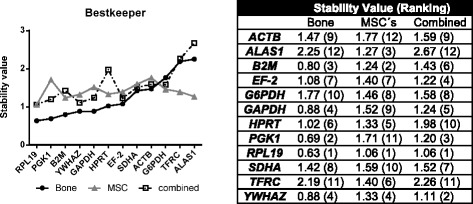
Ranking of candidate reference genes by Bestkeeper algorithm. Candidate reference genes were ranked by their stability value, as calculated by Bestkeeper. MSCs were both, osteogenic differentiated and control cells from ovMSCs at different time points (day 0, 7, 14, 21, and 28). Bone samples related to a pool of all experimental groups (control, OVX, OVXD and OVXDS). These samples were used for the consecutive arrangement of the graph

The Bestkeeper algorithm detected *RPL19* (stability value = 0.63) and *PGK1* (0.69) as stably expressed reference genes in ovine bone (Fig. 3).

However, in osteogenic differentiated ovMSCs *RPL19* (1.06) and *B2M* (1.24) revealed the highest stability. In the combined group the results of the Bestkeeper analysis showed that also *RPL19* with a stability value of 1.06 and additionally *YWHAZ* (1.11) were determined as the most stably expressed housekeeper genes.

### Normfinder calculation for stable reference genes

**Fig. 4:**
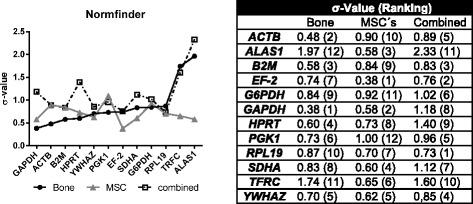
Ranking of candidate Reference gene by the Normfinder program. Candidate reference genes were ranked by their stability value (σ-value), as calculated by the Normfinder program. MSCs were both, osteogenically differentiated and control cells from ovMSCs at different time points (day 0, 7, 14, 21, and 28). Bone samples related to a pool of all experimental groups (control, OVX, OVXD and OVXDS). These samples were used for the consecutive arrangement of the graph

Inter- and intra-group expression variation was analyzed by using Normfinder (Fig. 4). It revealed that in bone tissue *GAPDH* (σ-Value = 0.38) followed by *ACTB* (0.48) were expressed on stable levels.

*EF-2* (0.38) and *GAPDH* (0.58) revealed the best stability in ovMSCs under osteogenic differentiation conditions. Combining the expression data from ovine bone and cultured ovMSCs, the results confirmed that *RPL19* and *EF-2* were the most stably expressed reference candidate genes.

### GeNorm determination of stable reference genes

By using GeNorm, *B2M* and *PGK1* (M-Value 0.48) were found to be stable reference genes in ovine bone samples while in ovine MSC constant expression values were determined for *ALAS1* and *EF-2* (M-Value 0.38).

**Fig. 5:**
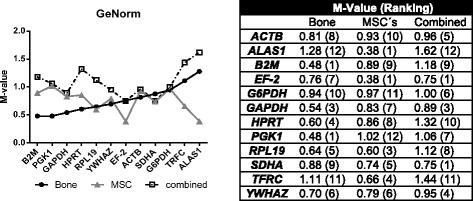
Ranking of candidate Reference gene by GeNorm. Candidate reference genes were ranked by their stability value (M-value), as calculated by GeNorm. MSCs were both, osteogenic differentiated and control cells from ovMSCs at different time points (day 0, 7, 14, 21, and 28). Bone samples related to a pool of all experimental groups (control, OVX, OVXD and OVXDS). These samples were used for the consecutive arrangement of the graph

The third group with combined data sets of bone and ovMSCs displayed that *EF-2* and *SDHA* (M-Value 0.75) have most stable gene expression (Fig. 5).

### Comprehensive ranking

**Fig. 6:**
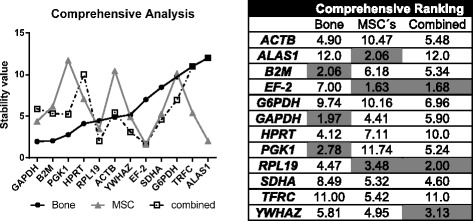
Comprehensive Analysis of all candidate reference genes. Candidate reference genes were ranked by their comprehensive ranking, as calculated by the RefFinder program. MSCs were both, osteogenic differentiated and control cells from ovMSCs at different time points (day 0, 7, 14, 21, and 28). Bone samples related to a pool of all experimental groups (control, OVX, OVXD and OVXDS). These samples were used for the consecutive arrangement of the graph

Finally, RefFinder tool was used to calculate the geometric mean of the ranking of all candidate genes. Results are shown in Fig. 6. In ovine bone tissue *GAPDH*, *B2M* and *PGK1* were the most stably expressed genes.

For ovine MSCs under osteogenic conditions *EF-2*, *ALAS1* and *RPL19* were the most suitable housekeeper genes. Combination of these two groups revealed that *EF-2*, *RPL19* and *YWHAZ* were stably expressed genes in both ovine bone and osteogenically differentiated ovMSCs.

### Gene ontology

After screening and validation of reference genes, four main reference gene candidates were obtained: *RPL19*, *B2M*, *GAPDH*, and *YWHAZ*. *RPL19* as part of ribosome plays an important role during protein synthesis process, *B2M* encodes a crucial protein component of class 1 major histocompatibility complex. *GAPDH* is a multifunctional gene which plays an important role in cell differentiation and cell pathologies, while *YWHAZ* is involved in regulating insulin sensitivity.

**Fig. 7:**
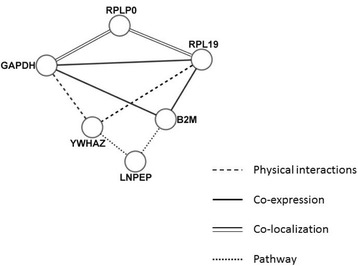
Network analysis correlated reference genes depending upon their physical interactions, co-expression, co-localization, and pathways. To understand the interactions among reference genes, network analysis was performed using GeneMANIA and Cytoscape. The bioinformatics approach to identify the relation between different reference genes showed no gene co-regulation, thereby making them suitable for the current study

Gene ontology showed the involved biological process, molecular function, and cellular process for each reference gene (Fig. 7). All four reference genes showed different gene ontologies. Network analysis showed the correlation between reference genes. The genes were correlated based on physical interaction, co-expression, co-localization, pathway, shared protein domains, and genetic interactions (Fig. 7). The reference genes were analysed according to human genome on GeneMANIA to predict interactions. *YWHAZ*, *GAPDH*, and *RPL19* shared physical interaction, while *B2M* showed no physical interaction with any of the reference genes. Furthermore, *GAPDH*, *RPL19*, and *B2M* were co-expressed with each other. Also, *GAPDH* and *RPL19* were co-localized via ribosomal protein lateral stalk subunit P0 (*Rplp0*). Gene ontology of *RPLP0* showed its role as a structural constituent of ribosomes. *YWHAZ* and *B2M* showed pathway interaction via leucyl and cystinyl aminopeptidase (*LNPEP*). *LNPEP* plays a role in maintaining homeostasis via peptide degradation. No reference gene shared genetic interactions and common protein domains.

## Discussion

RT-qPCR is still widely used for measuring expression of target genes, due to its high sensitivity, specificity, and low cost. Because of the potential variation of regulation of reference genes for RT-qPCR in different tissues and experimental conditions, it is important to validate the most stably expressed reference genes in order to obtain reproducible results. However, published data focusing on supposedly stably expressed reference genes showed that some of these were regulated differentially in various species and tissues [22]. Furthermore, only a few studies investigated the sheep as model organism [23].

Previous studies in sheep have described *SDHA* and *YWHAZ* as stably expressed genes in ovine peripheral whole blood [8]. The group of Vorachek et al. analyzed stably expressed reference genes in ovine neutrophils from healthy and foot rot affected sheep. They found *SDHA* and *G6PDH* as the best pair in healthy control sheep, whereas the best reference genes for food rot disease sheep were *GAPDH* and *YWHAZ* [7]. It is also described that *GAPDH* and *SDHA* should be stably expressed in ovine cerebrum and spleen, and *SDHA* and *RPL19* in the mesenteric lymph nodes [9]. A more recent work also looked for suitable reference genes in samples from the mandibular condyle both normal and fractured. The authors determined *RPL19*, *ACTB* and *PGK1* as the most stably expressed reference genes in both groups [12]. However, mandibular bone is different from trabecular bone in the spine and long bone where most osteoporotic fractures occur. Therefore, we used bone samples from our ovine osteoporosis model (control, OVX, OVXD & OVXDS) and ovine MSCs in vitro differentiated into osteoblasts. The special challenge was to find a set of reference gene that could be applied as well for analyses of in vivo ovine bone samples as for in vitro differentiates ovMSCs.

In the present study the expression stability of twelve potential reference genes was examined. Data analysis was performed using the web-based program RefFinder [14] identifying *GAPDH* and *B2M* as the most stable reference genes in bone samples from sheep. No clear overlap could be found between bone tissue and osteogenically differentiated ovMSCs. This may be caused by different cellular compositions or differences due to the in vitro osteogenic differentiation process of ovMSCs compared to in vivo bone samples. In ovMSCs, *EF-2* and *ALAS1* were the most stable reference genes, while *ALAS1* was the least stable gene in bone tissue and in the combined group. After we combined the Ct values of these two groups, RefFinder determined *EF-2*, *RPL19* and *YWHAZ* as most suitable reference genes. Bestkeeper algorithm calculated *RPL19* in all three cases as the best control gene. *TFRC* and *ALAS1* were unsuitable reference genes because the Ct values revealed huge variance especially in bone tissue. Due to this high variation of suitable reference genes in ovine bone samples and ovMSCs, we recommend the use of a combination of four reference genes for relative quantification, i.e. *B2M* and *GAPDH* for samples from ovine bone and additionally *RPL19* and *YWHAZ* (best of the combined group). *EF-2* was not included because the variation in the analysis of bone samples was very high. Network analysis showed the correlation between reference genes. The co-expression but not co-regulation of selected genes between the groups supports their deduction as reference genes alternatively or individually.

## Conclusion

In conclusion, this study demonstrates the importance of applying a set of reference genes for RT-qPCR analysis in sheep on a tissue or cell type specific basis. Based on our data we recommend testing of *B2M*, *GAPDH*, *RPL19*, and *YWHAZ* for relative quantification of gene expression studies in ovine bone which it is a robust bio-medical model for evaluating bone-substituents in osteoporosis.

## Additional files

Additional file 1: Control of osteogenic MSC differentiation by AlizarinRed S staining. MSCs from ovine origin were osteogenically differentiated for indicated time points and stained by AlizarinRed S. Red color shows calcium deposits which suggest osteogenic differentiation. (PDF 1057 kb)

Additional file 2: BioGPS Analysis of tested reference genes. BioGPS analysis revealed ubiquitous expression of all reference genes in the tissues validated of BioGPS. (PDF 178 kb)

## Abbreviations

FDA: Food and Drug Administration
MSC: Mesenchymal stromal cells
Ov: Ovine
OVX: Ovariectomy
OVXD: Ovariectomy + calcium and vitamin low diet
OVXDX: Ovariectomy + calcium and vitamin low diet + steroids
RT-qPCR: Quantitative real time PCR
