# Supplementary figures and images for: A tissue-based approach to selection of reference genes for quantitative real-time PCR in a sheep osteoporosis model
Source: BMC Genomics. 2017 Dec 19;18:975. doi: 10.1186/s12864-017-4356-4 (PMC5735898; doi:10.1186/s12864-017-4356-4)

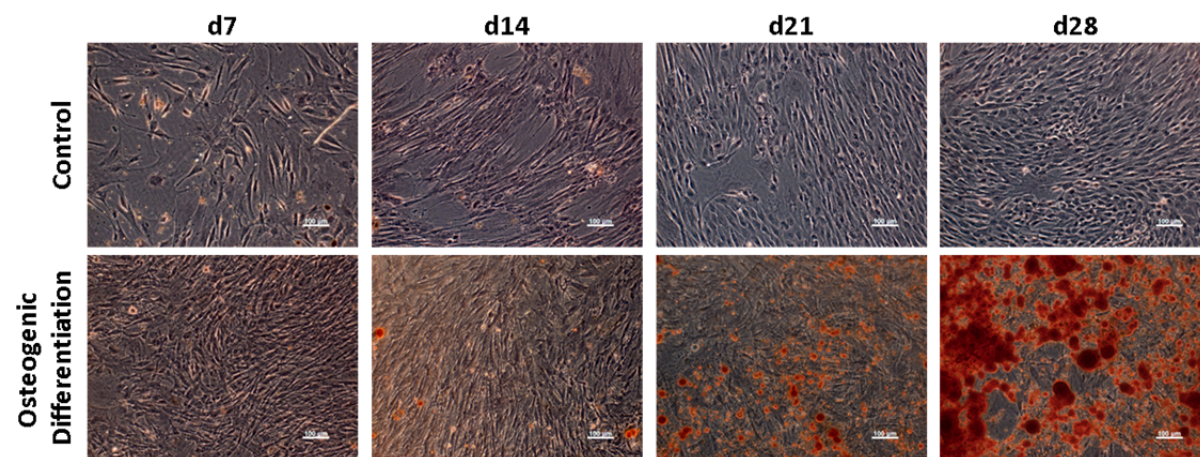

Supplement: Supplementary file 1 — Control of osteogenic MSC differentiation by AlizarinRed S staining. MSCs from ovine origin were osteogenically differentiated for indicated time points and stained by AlizarinRed S. Red color shows calcium deposits which suggest osteogenic differentiation. (PDF 1057 kb) [file 12864_2017_4356_MOESM1_ESM.pdf]

## BioGPS Analysis

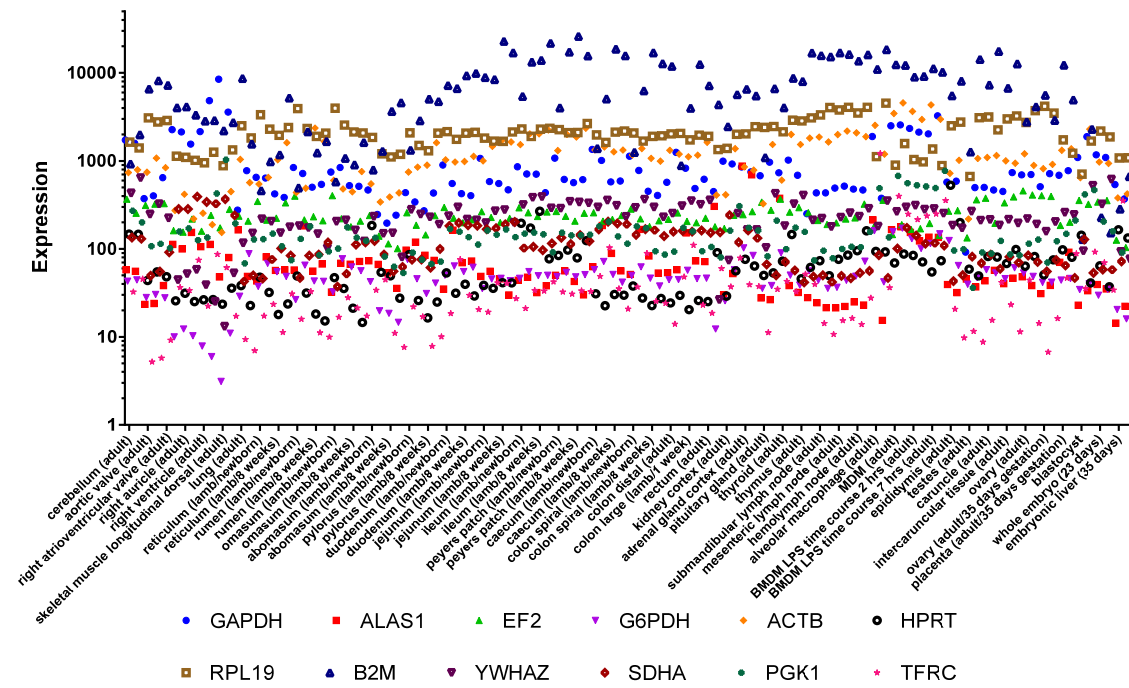

Supplement: Supplementary file 2 — BioGPS Analysis of tested reference genes. BioGPS analysis revealed ubiquitous expression of all reference genes in the tissues validated of BioGPS. (PDF 178 kb) [file 12864_2017_4356_MOESM2_ESM.pdf]
